# Nesting on Cell Phone Towers: An Inexplicable Breeding Strategy by Asian Woollynecks *Ciconia episcopus* in Bangladesh

**DOI:** 10.1002/ece3.71353

**Published:** 2025-04-21

**Authors:** Allama Shibli Sadik, Ashis Kumar Datta

**Affiliations:** ^1^ Forest Department Wildlife Center Gazipur Bangladesh; ^2^ Department of Zoology Jahangirnagar University Savar Bangladesh

**Keywords:** breeding biology, *Ciconia episcopus*, nest predation, nesting ecology

## Abstract

The Asian Woollynecks were once thought to be a rare winter migrant to the wetlands of Bangladesh, and until very recently, only a few incidents of nesting had been observed. New information shows expanding populations across their habitat, though little is known about their movement ecology, breeding ecology and preferences for artificial nest sites. In this paper, we documented some crucial nesting and breeding information on this species. We used camera traps as a means of passive monitoring and identified Large‐billed crow *Corvus macrorhynchos* and House crow *Corvus splendens* as potential nest predators. Three nests were found in artificial structures, where anthropogenic activity, like disturbances during cell phone tower maintenance, or predators limit the breeding success of Asian Woollynecks.

## Graphical abstract

In this paper, we documented some crucial nesting and breeding information on Asian Wollynecks. We used camera traps as a means of passive monitoring and identified Large‐billed crow *Corvus macrorhynchos* and House crow *Corvus splendens* as potential nest predators.

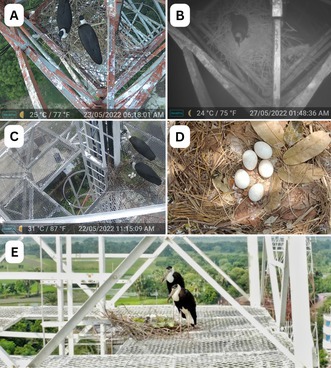

## 1 Introduction

Nesting in human‐modified environments and use of man‐made structures is a common phenomenon in storks. In a beneficial standpoint, such man‐made structures offer access to perches, roosting or breeding sites (Mainwaring 2015). However, electrocution and collisions with powerlines have been documented in many regions, and a long‐term study in Poland found no differences in breeding success of stork nests in natural and man‐made structures (Tryjanowski et al. 2009). An interesting study to detect the possible effect of electromagnetic fields on White Stork *Ciconia ciconia* productivity, Balmori (2005) in Spain found that microwaves had an impact on the reproduction of the white stork.

Asian Woollyneck *Ciconia episcopus* is a globally near threatened (NT) large wader present across South and South‐east Asian countries (Birdlife International 2020; Birdlife International 2023; Sundar 2020). Recent findings suggest that Woollyneck has established a stronghold in South Asia, particularly in Nepal, India and Sri Lanka, probably due to agricultural expansion, land use change and subsequent behavioural plasticity of the stork (Sundar 2020; Ghimire et al. 2021a, 2021b). Although the species was considered to be negatively influenced by human presence, new information shows that Asian Woolly‐necked Stork prefers natural and artificial wetlands, paddy fields, pastures, grasslands, man‐made urban structures, nesting on agro‐forestry‐related trees amid crop fields, and other agricultural landscapes (Sundar 2006; Choudhary et al. 2013; Vaghela et al. 2015; Greeshman et al. 2018; Kittur and Sundar 2021).

**FIGURE 1:**
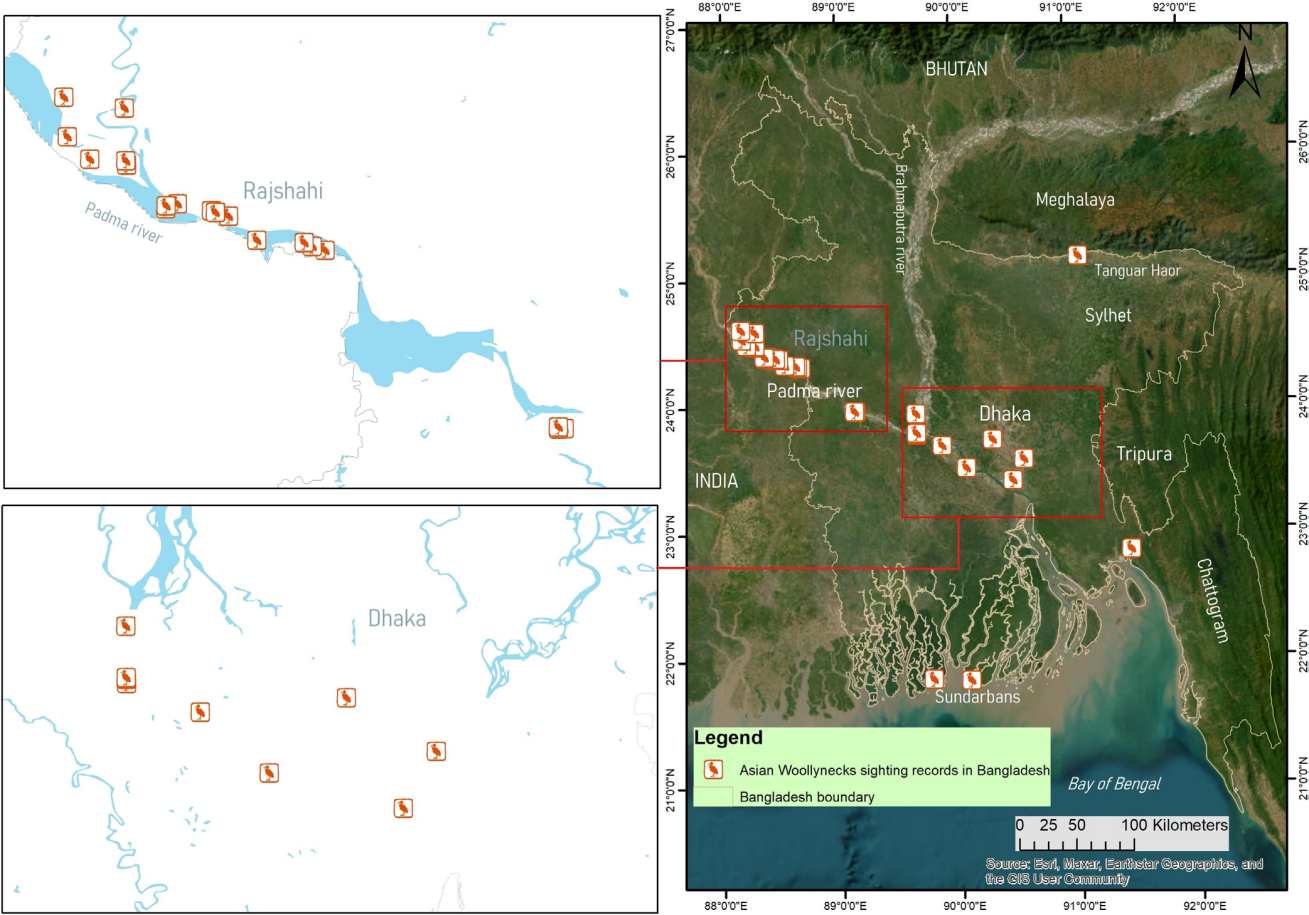
Recent observations of Asian Woollynecks in Bangladesh (Date sources: ebird 2021, and personal observations).

The species has a large population size and is likely stable or increasing across its range; however, the population in South‐east Asia has undergone considerable declines due to many anthropogenic activities (Birdlife International 2020; Kittur and Sundar 2020; Sundar 2020; Ghimire et al. 2021a; Ghimire et al. 2021b). The bird is threatened in South‐east Asian range countries; it appears stable or possibly expanding in the South Asian range, such as in India, Pakistan and Nepal (Kittur and Sundar 2020; Sundar 2020). The IUCN Red List has downlisted Asian Woollynecks from Vulnerable to Near Threatened in response to criticism over its sudden unsubstantiated uplisting alongside recent ecological data from across its range. In Bangladesh, this stork is considered a critically endangered (CR) rare winter migrant distributed mostly in landmasses resulting from the accretion of sediments (locally known as chars) and wetlands of the Rajshahi Division (along Padma River) and mud‐banks of the northeast region of the country (IUCN Bangladesh 2015). Only a handful of observations were recorded till 2013; since then, sightings of Woollynecks are now reported sporadically in Padma riverine areas (ebird 2021; Figure 1). However, we still lack population data. There were few anecdotal breeding records of woolly‐necked storks in Bangladesh (Khan 1987), until recently, Hasan and Ghimire (2020) confirmed the first breeding of this species from the Rajshahi and Chapainawabganj districts. Herein, we present additional breeding records from a recent survey alongside detailed observations on nests to understand aspects, such as nesting ecology, human disturbances and nest predation.

### 1.1 Study Area

**FIGURE 2:**
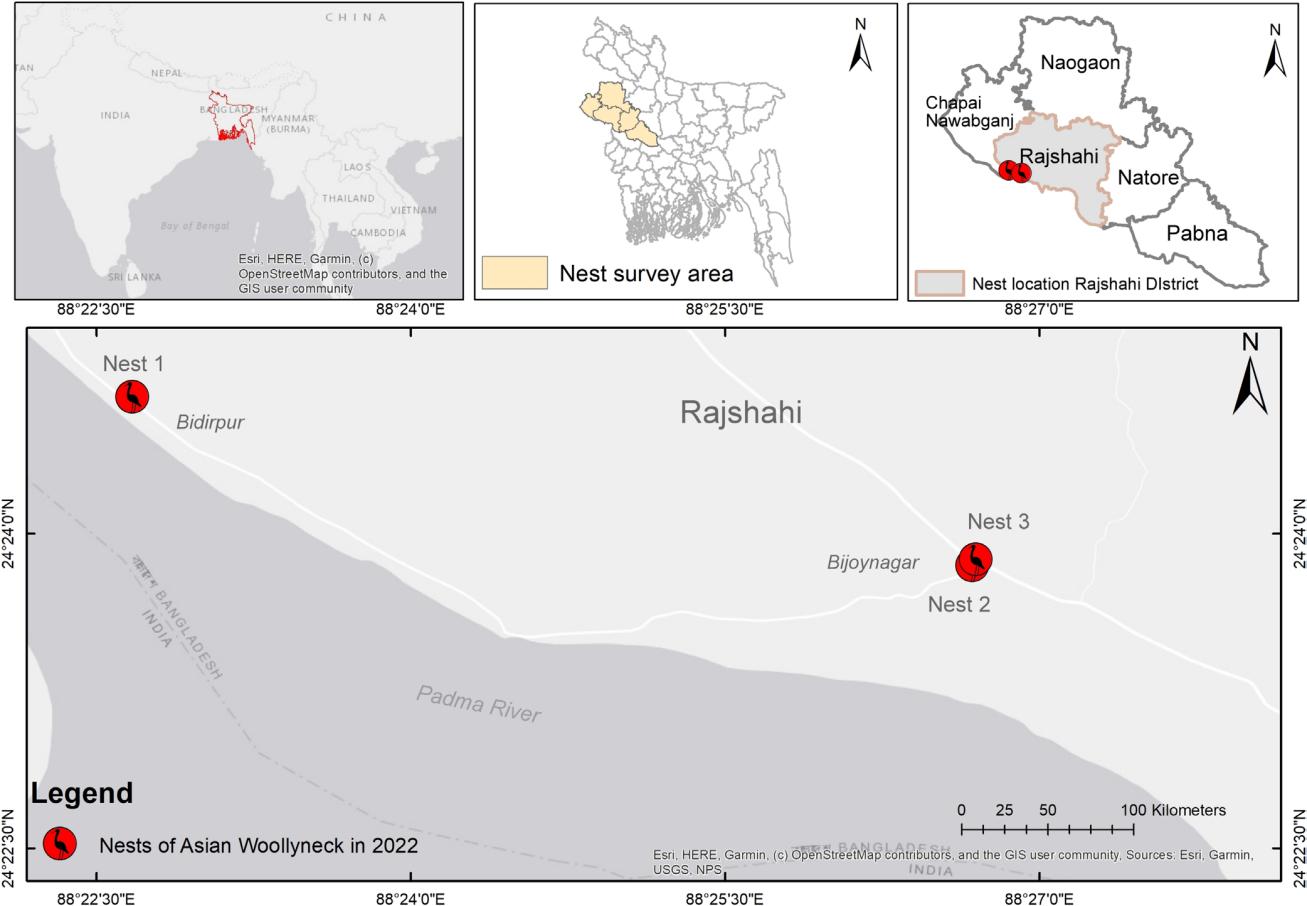
Map showing study area and confirmed breeding sites of Asian Woollyneck in Rajshahi districts, Bangladesh.

Based on published information of Woollynecks nesting on large and medium‐sized trees, man‐made or urban structures, such as cell phone towers (Vaghela et al. 2015; Hasan and Ghimire 2020; Kittur and Sundar 2021), we surveyed five districts viz. Pabna, Natore (Chalon beel area), Rajshahi, Chapainawabganj and part of Naogaon for potential nesting sites (Figure 2). Except Naogaon, all four districts are situated on the banks of the river Padma, the distributary of the river Ganges in Bangladesh. Large waders can be observed in seasonally accreted landmasses, small channels, grasslands and further upstream along the Padma in these districts during winter. The waders include Painted Stork *Mycteria leucocephala* , Black Stork *Ciconia nigra* , Asian Openbill *Anastomus oscitans* , Asian Woollyneck Stork and large multi‐species congregations of migratory waterfowl (Thompson 2021).

## 2 Methods

The study was carried out from 5 October 2021 to 30 October 2023. We actively searched for possible nesting sites by walking, engine boat or by motor bike as feasible to inspect cell phone towers. The vast majority of the study area was urban, with patches of more natural habitat, including seasonally accreted lands, riverbanks, homestead plantations, agricultural fields and especially man‐made structures like buildings and cell phone towers.

Surveys included the sites where Woollyneck storks had nested in previous years (Hasan and Ghimire 2020). To obtain a more comprehensive coverage of stork nesting on cell phone towers, we consulted three major mobile network provider companies, viz., Teletalk, Banglalink and Robi, who altogether maintain 990 towers in our survey areas. Instead of physically inspecting the tops of all the towers, we interviewed people living around the towers and cell phone tower maintenance crew to document potential nesting sites of Asian Woollynecks. Once nest building was completed, we installed cameras (camera model: GardePro) for an average of 74 days on two nests. Camera traps were installed on the nests with assistance from cell phone tower maintenance crews. They were given hands‐on training on installing and operating camera traps prior to that. Camera information was used alongside direct visual observation (using binoculars) and by drone (used once for a single nest to avoid disturbances). All breeding activities were documented with minimal disturbances. During the survey, we conducted focus group discussions and interviews with local community members. The sole purpose of these interviews was to find nesting information and their perception on conserving colonial waterbirds, especially storks, in the study area. We did not collect any individual data to protect their identity.

**FIGURE 3:**
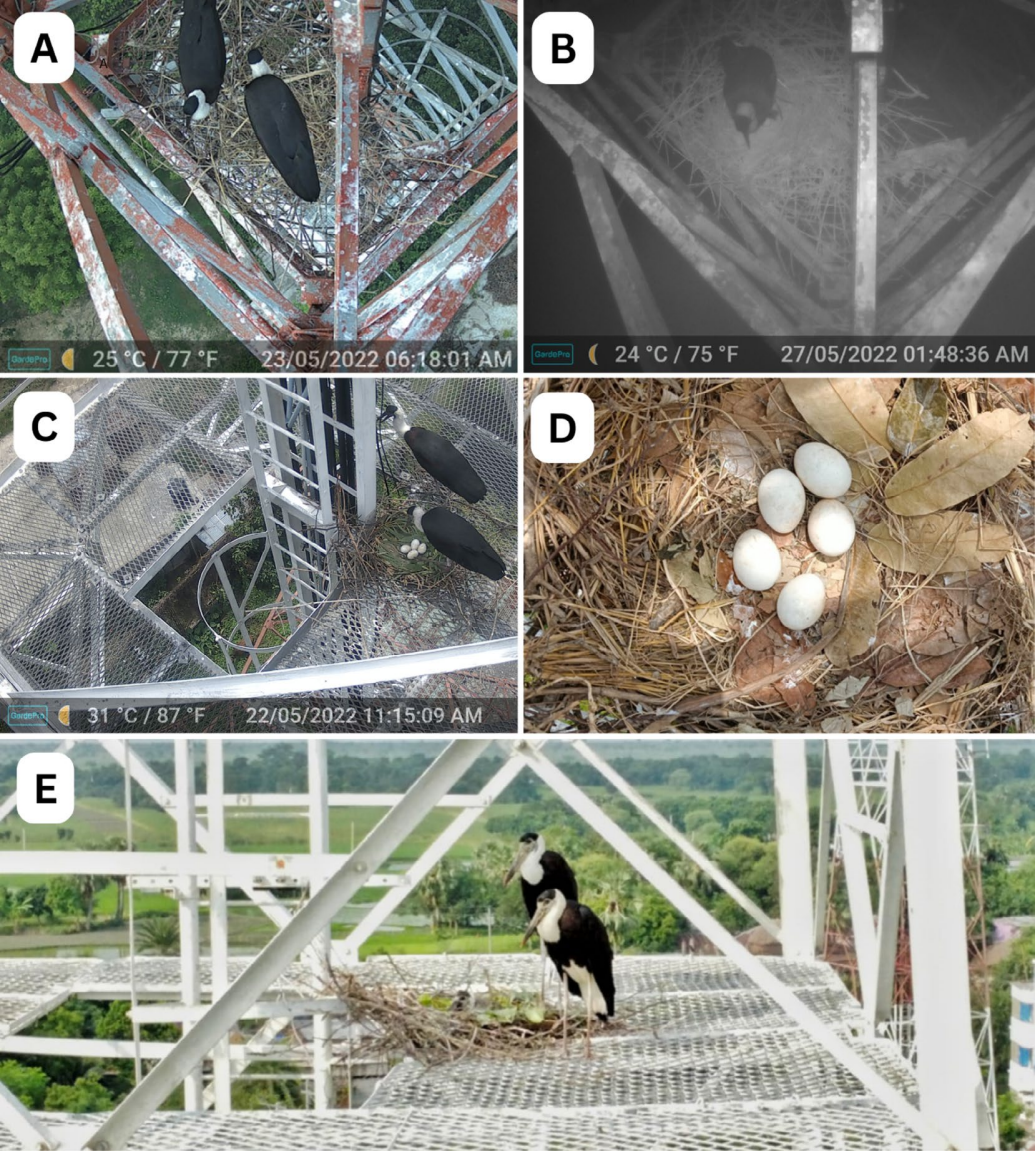
Camera trap and drone photograph of breeding activities of Asian Woollynecks in study area [(A) nest building by Woollyneck pair, (B) egg incubation, (C) Nest 2 with complete clutch of four eggs, (D) Nest 1 with five eggs, (E) Woollyneck pair with a chick at Nest 3].

A nest was defined as successful when it produced at least one fledgling. For other nests, nest failure was confirmed using eggshell (suggesting predation) or dead nestlings. Fledging success was calculated as the proportion of eggs in a clutch that survived to fledging (Cornell et al. 2011). Nest building materials were identified after the birds left the nests and from photographs taken using the camera traps (Figure 3). Due to small sample size, we did not perform any statistical tests but provided descriptive summaries. The study area map was produced by ArcGIS 10.3.

## 3 Results

After surveying over 230 km throughout the study area (Figure 1), we observed eight Asian Woollynecks in 2022 and six in 2023. Three nests of Asian Woollynecks were found during 2022, and all of these nests were built on cell phone towers (Figure 2). Nest 1 and Nest 3 were newly described nesting sites. Nest 2 in the Bijoynagar area has been known to have Asian Woollynecks nesting in the past. Nest 1 was predated by a Large‐billed Crow *Corvus macrorhynchos* . In between egg laying and predation, the Crows were recorded six times. On 2 July 2022, the tower maintenance (welding) started at 11:57 AM and lasted till 1:43 PM, where the predation event was recorded at 12:05 PM.

Likewise, Nest 2 was abandoned with eggs predated by a House Crow *Corvus splendens* . Before the predation event took place on 26 May 2022, at 2:26 PM, the House crow was seen at least 10 times near nests. Both nesting pairs appeared in the towers several times after predation events. Afterwards, the pair from Nest 2 relocated to Nest 3, which was 60 m away, and laid a single egg. The pair also moved nesting materials from Nest 2 to Nest 3. One egg in Nest 3 successfully hatched and fledged during the 2022 breeding season (Figure 3E). The clutch sizes of the three nests were five, four and one.

**FIGURE 4:**
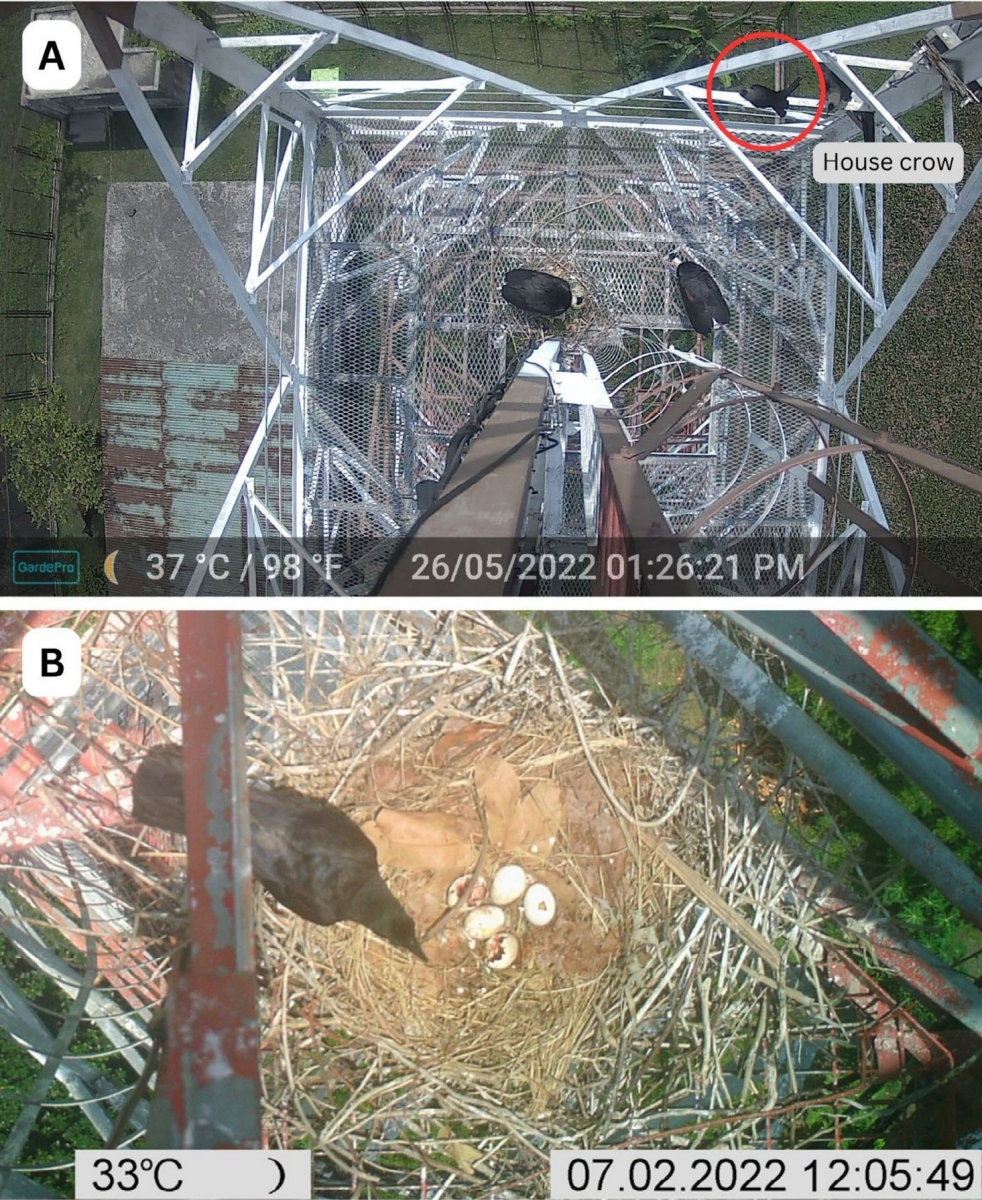
Nest predation attempt [(A) by House Crow] and nest predation [(B) by Large‐billed Crow] of Asian Woollynecks in Bangladesh.

We find that our observed Nest 1 and Nest 2 were distressed because of the frequent presence of maintenance crews of the tower. The breeding pair was away from the nest for a few hours because of maintenance workers. Both the nests were abandoned and subsequently predated by House Crow and Large‐billed Crows, respectively. Our camera trap photograph revealed the incidents (Figure 4). Additionally, the photograph showed that five eggs in Nest 1 were almost ready to hatch on the basis of the appearance of the dead hatchlings (Figure 4B).

In the 2023 breeding season, the pairs did not return to their nesting tower. However, one pair started building a nest on the previously used Nest 3 in the Bijoynagar area. The nest was nearly completed in the middle of June 2023 but was destroyed during maintenance work on the cell phone tower on 16 June.

For nesting, Asian Woollynecks used green leaves and sticks of *Acacia auriculiformis* , *Dalbergia sissoo* , *Swietenia macrophylla* , *Eucalyptus* sp., *Mangifera indica* and a few unidentified grass species. In Table 1, we have summarised all of the information related to the three observed nests for easy reading.

**TABLE 1:** Details of nest locations of three Asian Woollyneck nests in Bangladesh.

Nesting parameters	2022			2023		
Nest pumber	Nest 1	Nest 2	Nest 3	Nest 1	Nest 2	Nest 3
Nest location	In a paddy field at Bidirpur Bazar	Inside cultivable land at Bijoynagar	In a paddy field at Bijoynagar	No nest found	No nest found	In a paddy field at Bijoynagar
Onset of nest building	3 March 2022	22 April 2022	26 may (carried nest materials from Nest 2)	—	—	Tower demolished and nest destroyed
Substrate/tree	Cell phone tower	Cell phone tower	Cell phone tower	—	—	Cell phone tower
Height from ground (m)	52	42	42	—	—	42
Distance to nearest road (m)	20	25	10	—	—	10
Distance to nearest Tree (> 10 m)	50	300	300	—	—	300
Distance to Padma River m	330	1146	1160	—	—	1160
Nest diameter (total length) in cm	63.5	61.8	Not measured	—	—	Not measured
Nest diameter (core area) in cm	45.5	43.4	Not measured	—	—	Not measured
Breeding parameters						
Date of laying of first egg	27 May 2022	21 May 2022	26 May 2022	—	—	—
Last egg laid	6 June 2022	Not known	Not known	—	—	—
Clutch size	5	4	1	—	—	—
Incubation period	Not completed	Not completed	—	—	—	—
Nest predation/egg lost	5	4	None	—	—	—
Nest fate	Predated (2 July 2022)	Abandoned and predated	Successfully fledged	—	—	—

## 4 Discussion

During our 2‐year breeding survey, where we covered an extensive area over two consecutive years, we found very few nests, and all of these were on cell phone towers. The previous study in Bangladesh by Hasan and Ghimire (2020) also observed nests only on cell phone towers. Use of mobile towers for nesting by Asian Woollynecks has been observed in several locations (Vaghela et al. 2015; Hasan and Ghimire 2020; Sundar 2020; Kittur and Sundar 2021). Most of these studies provide anecdotal observation and have surmised that storks use towers for increased visibility, height, avoidance of human disturbances and stronger nest substrates. However, the extensive study on breeding ecology by Kittur and Sundar (2021) showed use of artificial substrates (electricity pylons) in an agriculture‐dominated landscape by Asian Woollynecks to be minimal (8%–13% of nests each year). The study area lacks larger trees; however, the urbanised landscape is predominantly agricultural, along with larger waterbodies. Clearly, the use of artificial sites appears to be a behaviour regulated by local conditions, and it is not clear why the storks in Bangladesh are using cell phone towers over trees all the time.

We observed that Asian Woollynecks initiated nesting in April or May, which is later relative to previous nesting records in Bangladesh (September—November; Hasan and Ghimire 2020) but overlapped with observations in India (May—October; Kittur and Sundar 2021). In the lowlands of Nepal, the onset of breeding is observed from March to November (spanning summer‐monsoon) where the nesting period coincides with the monsoon (Ghimire et al. 2022). Most studies on Asian Woollynecks are short‐term, making it difficult to know for sure the nesting period, and the only multi‐year study in north India showed that the nesting period was consistently between May and October (Kittur and Sundar 2021), and our observations over 2 years also show some inter‐annual consistency of nesting with nests observed between April and May (this study). With the available information, it appears that there is a variation in the nesting season of the Asian Woollynecks across its range.

Deposition of green plant material in a nest is a quite common in birds, but their function is not properly known, as in the case of Asian Woollynecks in this study. Similar to Woollynecks, other stork species, such as Asian Openbill ( *Anastomus oscitans* ) and Wood Stork ( *Mycteria americana* ) are also seen to use green nesting materials to line the inner surface of the nest (Rodgers Jr et al. 1988). It is believed that green Eucalyptus trees have volatile chemicals that are known to repel or even kill ectoparasites (Gwinner et al. 2000). However, in the case of Wood Stork ( *Mycteria americana* ) role of green leaves in repelling nest ectoparasites was not evident (Rodgers Jr et al. 1988). Clutch sizes of Asian Woollynecks in north India, determined from fledged chicks, were 3–5 (average of 3.1), with one pair fledging six chicks (Sundar 2020; Kittur and Sundar 2021). Our findings of a single egg in a nest and the subsequent fledging of the hatchling from this egg are unusual.

The primary reason for nest disturbance and abandonment that led to egg predation was due to disturbance caused by maintenance workers. Nest abandonment due to mobile tower maintenance workers was also reported from the same study area (Hasan and Ghimire 2020). We believe this to also be the reason why storks avoided nesting on the towers in 2023. Asian Woollyneck showed high nest site fidelity in north India despite year‐long human presence (44% of 166 unique sites were used more than once; Kittur and Sundar 2021) (Kittur and Sundar 2021). In Bangladesh, Asian Woollynecks abandoned nest sites after disturbance by humans, as Hasan and Ghimire (2020) reported, and our observations also indicate the same. Observations in Bangladesh suggest that Asian Woollynecks face enormous disturbances when nesting on cell phone towers. It is unclear, therefore, why all the nests of this species found in Bangladesh are on cell phone towers. Such continued disturbance by humans is likely to cause storks to start nesting on trees where regular human visits are unlikely, except perhaps by hunters. The contradictory situation of very high nest disturbance by humans on cell phone towers and no nests being found on trees is, as yet, inexplicable. Ghimire et al. (2020) identified Yellow‐throated Martin *Martes flavigula* , a mammalian nest predator for Asian Woollyneck in Nepal; in this study, we identified large‐billed crow *Corvus macrorhynchos* and House crow *Corvus splendens* as competent nest predators.

Habitat and nest protection is suggested to be a likely requirement for Asian Woollyneck survival in South‐East Asia (Birdlife International 2023). Protecting nests in cell phone towers during the breeding season appears to be a critical requirement in Bangladesh as well. Working with cell phone providers to understand how workers can minimise disturbances during the Asian Woollyneck breeding season is an important requirement. The study concludes that studies on the habitat suitability analysis, long‐term viability of nesting on cell phone towers, the role of electromagnetic radiation, and predator–prey interactions in these habitats should be carried out immediately to conserve Asian Woollynecks in Bangladesh.

## Author Contributions

**Allama Shibli Sadik:** conceptualization (equal), data curation (supporting), funding acquisition (lead), investigation (equal), methodology (equal), project administration (lead), writing – original draft (supporting). **Ashis Kumar Datta:** data curation (equal), formal analysis (lead), methodology (equal), supervision (equal), writing – original draft (lead), writing – review and editing (lead).

## Conflicts of Interest

The authors declare no conflicts of interest.

## Supporting information


**Data S1.** Details of nesting parameters of three Asian Woollyneck nests in Bangladesh.

## Data Availability

Data collected for this research are available in Table 1 of this manuscript.
