# Supplementary material for: Nesting on Cell Phone Towers: An Inexplicable Breeding Strategy by Asian Woollynecks Ciconia episcopus in Bangladesh
Source: Ecol Evol. 2025 Apr 21;15(4):e71353. doi: 10.1002/ece3.71353 (PMC12011550; doi:10.1002/ece3.71353)
Supplement: Supplementary file 1 — Data S1. Details of nesting parameters of three Asian Woollyneck nests in Bangladesh. [file ECE3-15-e71353-s001.docx]

Details of nesting parameters of three Asian Woollyneck nests in Bangladesh.

| Nesting parameters | 2022 | | | 2023 | | | | | | |
| --- | --- | --- | --- | --- | --- | --- | --- | --- | --- | --- |
| Nest pumber | Nest 1 | Nest 2 | Nest 3 | Nest 1 | | Nest 2 | | Nest 3 | |  |
| Nest location | In a paddy field at Bidirpur Bazar | Inside cultivable land at Bijoynagar | In a paddy field at Bijoynagar | No nest found | | No nest found | | In a paddy field at Bijoynagar | |  |
| Onset of nest building | 3 March 2022 | 22 April 2022 | 26 may (carried nest materials from nest 2) | - | | - | | Tower demolished and nest destroyed | |  |
| Substate/ tree | Cell Phone tower | Cell Phone tower | Cell Phone tower | - | | - | | Cell Phone tower | |  |
| Height from ground (m) | 52 | 42 | 42 | - | | - | | 42 | |  |
| Distance to nearest road (m) | 20 | 25 | 10 | - | | - | | 10 | |  |
| Distance to nearest Tree (>10m) | 50 | 300 | 300 | - | | - | | 300 | |  |
| Distance to Padma River m | 330 | 1146 | 1160 | - | | - | | 1160 | |  |
| Nest diameter (total length) in cm | 63.5 | 61.8 | Not measured | - | | - | | Not measured | |  |
| Nest diameter (core area) in cm | 45.5 | 43.4 | Not measured | - | | - | | Not measured | |  |
| Breeding Parameters |  |  |  |  |  | |  | |  |  |
| Date of laying of first egg | 27 May 2022 | 21 May 2022 | 26 May 2022 | - | | - | | - | |  |
| Last egg laid | 6 June 2022 | Not known | Not known | - | | - | | - | |  |
| Clutch size | 5 | 4 | 1 | - | | - | | - | |  |
| Incubation period | Not completed | Not completed | - | - | | - | | - | |  |
| Nest predation/egg lost | 5 | 4 | none | - | | - | | - | |  |
| Nest fate | Predated  (1 July 2022) | Abandoned and predated | Successfully fledged | - | | - | | - | |  |
